# Application effect of problem-based learning teaching model in respiratory critical medicine

**DOI:** 10.3389/fmed.2025.1648744

**Published:** 2025-11-21

**Authors:** Ju Jiang, Haoda Yu, Caixia Hu, Huiya Zhou, Xiaodong Cao

**Affiliations:** 1 Department of Respiratory and Critical Care Medicine, Affiliated Wuxi People's Hospital of Nanjing Medical University, Wuxi, Jiangsu, China; 2 Department of Nursing, Affiliated Wuxi People's Hospital of Nanjing Medical University, Wuxi, Jiangsu, China

**Keywords:** respiratory critical medicine, problem-based learning, teaching method, probiotics, early enteral nutrition, intestinal adaptability

## Abstract

**Objective:**

To explore the application effect of problem-based learning (PBL) teaching model in the teaching of respiratory critical medicine.

**Methods:**

A total of 114 clinical medicine students who had internships in our hospital from January 2021 and December 2023 selected as the research participants. These students were randomly divided into a PBL group (*n* = 57) and a lecture-based learning (LBL) group (*n* = 57). The PBL group received PBL teaching method while the LBL group received LBL teaching method. The teaching effect and student satisfaction of the two groups were compared.

**Results:**

Compared with the LBL group, the PBL group performed better in clinical operations, theoretical knowledge, case analysis, and student satisfaction.

**Conclusion:**

The PBL teaching method is conducive to improving the teaching effectiveness and increasing the satisfaction of students. It has high promotion and application value.

## Introduction

Respiratory medicine, as a key branch of clinical medicine, is highly theoretical, practical and applicable. It holds an indispensable position in the medical teaching system and has extensive application value. It not only covers a wealth of basic theoretical knowledge such as anatomy, physiology and pathology, but also involves the diagnosis, treatment and prevention strategies of numerous complex diseases. It is closely intertwined with multiple clinical disciplines and has always been an important and challenging discipline in the field of clinical medicine (1). With the rapid development of medical technology and the continuous changes in the clinical disease spectrum, new types and treatment methods of respiratory system diseases are emerging one after another. This poses higher requirements for the teaching of respiratory medicine (2). How to enable medical students and interns to better master the knowledge and skills of respiratory medicine and adapt to the constantly changing clinical needs has become an important issue that current medical education urgently needs to address.

Teaching methods, as a core factor influencing teaching quality, play a crucial role in the transmission of knowledge and the cultivation of skills. To better assist in the implementation of respiratory system teaching, it is particularly crucial to find an effective teaching method for the respiratory system. This is of profound significance for improving the teaching quality of respiratory medicine and cultivating medical professionals with solid professional knowledge and outstanding clinical skills (3).

In the past, the traditional lecture-based learning (LBL) teaching method dominated in medical education, and was widely used during the clinical practice of interns. The LBL teaching method is teacher-centered, and through systematic classroom lectures, the theoretical knowledge in the textbooks is imparted to students step by step. This teaching approach can, to a certain extent, help students build a complete knowledge system and has played an important role in the rapid dissemination of medical knowledge (4). However, with the update of medical education concepts and the change of clinical practice demands, the limitations of the LBL teaching method have become increasingly evident. It overly focuses on the imparting of theoretical knowledge, often being confined to textbook content and lacking close integration with clinical practical situations. During the teaching process, students are mostly in a passive state of accepting knowledge, lacking opportunities for active thinking and exploration, which makes it difficult for them to effectively exercise their clinical thinking abilities and practical operation skills. Long-term reliance on this teaching method leads to interns often showing problems such as inflexible application of knowledge and insufficient clinical decision-making ability when facing real and complex clinical cases. As a result, their clinical practice abilities continue to decline, failing to meet the requirements of modern clinical medical work (5, 6).

The problem-based learning (PBL) teaching method was initially proposed by Professor Barrows from the United States. As an innovative educational model, it has gradually gained widespread attention and application in the global medical education field. This method mainly refers to a teaching model that is student-centered, led by teachers, and based on patients’ disease issues (7). In PBL teaching, teachers meticulously design a series of real-life problems closely related to clinical practice. They guide students to work in groups and encourage them to independently research literature, analyze problems, discuss and communicate to actively explore solutions to the problems. During this process, students no longer serve as passive containers for knowledge but become active participants in learning. They can fully exert their own initiative, think actively, and be courageous in questioning, thereby effectively cultivating their independent thinking ability, teamwork ability, communication and expression ability, as well as lifelong learning ability (8). Numerous studies have shown that the PBL teaching method has achieved remarkable results in improving students’ problem-solving abilities and autonomous learning skills (9, 10). However, the application effect of PBL teaching model in the teaching of respiratory critical medicine is not clear.

In our research, we aimed to deeply explore the application effect of the PBL teaching model in respiratory critical care medicine teaching. By comparing the differences between the PBL teaching model and the traditional LBL teaching model, we evaluated the effectiveness of the PBL teaching model in enhancing students’ basic knowledge and practical skills, providing scientific basis and practical references for the reform and optimization of respiratory critical care medicine teaching methods.

## Data and methods

### General data

A total of 114 clinical medicine students who had internships in our hospital from January 2021 and December 2023 selected as the research participants. All students signed the informed consent form for this research. This study was approved by the ethics committee of Affiliated Wuxi People’s Hospital of Nanjing Medical University, and the approval number was KY24038.

### Sample size calculation

Power analysis was carried out in this study using G*Power 3.1.9.7 software to determine the sample size required to detect statistical differences. With an alpha level of 0.05 and 80% power, the research revealed that a sample size of 45 medicine students per group was required. Considering the possibility of data loss and participant withdrawals during the research process, to ensure an adequate sample size, the sample size of each group was increased by 20%. Therefore, to draw reliable conclusions, the study sample sizes were 57 medicine students per group.

### Randomization

This study employed a computer-generated random number table for random grouping. The specific procedure was as follows: Using professional statistical software (SPSS 20.0), 114 random numbers were set to be generated within the range of 1–114. After generating the random numbers, they were sorted according to their size, and the first 57 numbers corresponded to the students assigned to the PBL group, while the last 57 numbers corresponded to the students assigned to the LBL group. This method based on the computer-generated random number table ensures that each student has an equal probability of being assigned to either group, thereby guaranteeing the randomness and scientific nature of the grouping process.

To maintain the confidentiality of the allocation, the random grouping process was carried out by an independent researcher who was not involved in the research. After generating the random numbers and completing the grouping, the researcher sealed the grouping results in an opaque envelope, with only the student’s number marked on the envelope. When the intern students were enrolled, another teaching manager who was not involved in the subsequent teaching and evaluation process opened the envelopes in sequence according to the numbers on the envelopes and informed the students of their assigned groups. Throughout the process, the personnel responsible for teaching implementation and effect evaluation were unaware of the specific grouping situation until the final data analysis stage, at which point the grouping information was revealed to avoid bias caused by knowing the grouping information in advance.

### Blinding

In this study, due to the uniqueness of the teaching model, it was difficult to implement a blind method for both students and the instructors. Students could clearly perceive whether they were receiving the PBL teaching method or the lecture-based learning method, and the instructors also knew which teaching model they were using for their lessons. However, to minimize bias as much as possible, this study adopted a blind assessment method. Specifically, the assessment of teaching effectiveness and the survey of student satisfaction were conducted by two senior experts in respiratory critical care medicine who were unrelated to the research implementation. These two experts only received the students’ assessment materials and questionnaires before the assessment and did not know the students’ groups. They independently scored the students’ learning effectiveness based on the pre-set scoring criteria and conducted statistical analysis of the students’ satisfaction questionnaires. Through this blind assessment method, it is possible to reduce the subjective bias caused by the assessment personnel knowing the students’ groupings and improve the objectivity and reliability of the assessment results.

## Methods

Students in the LBL group received LBL teaching method. The specific content was as follows: The main teaching content was respiratory critical diseases, and the teachers taught the interns about the pathogenic factors, clinical features, examination methods, differential diagnosis, treatment plans and complications of respiratory critical diseases.

Students in the PBL group received PBL teaching method. The specific content was as follows: (1) Basic knowledge teaching: The teacher taught in detail the basic knowledge of the epidemiological characteristics, clinical manifestations, treatment principles and complications of respiratory critical diseases. (2) Raise questions: According to the requirements of the clinical internal medicine teaching syllabus, the teacher extensively consulted the relevant data of cases, and selected a case 7 days before each class according to the course progress, and set corresponding questions based on the case situation and students’ ability. (3) Preparation before class: Interns were required to consult relevant knowledge through various learning tools according to the materials provided by the teachers, and combine with own knowledge reserves to sum up their own ideas to solve problems, and at the same time, have full discussion and analysis with other interns. (4) Summary stage: The teacher supplemented and revised the discussion content of the students, introduced the relevant new progress, and conducted a reasonable assessment of the students’ performance.

### Primary outcome

Teaching effect evaluation. After the end, the students’ basic knowledge and practical skills were assessed by written test and clinical operation. There were three items in total: clinical operation, theoretical knowledge and case analysis, and the full score of each item was 100 points. The higher the score, the better the teaching effect.

### Secondary outcome

Student satisfaction evaluation. The hospital’s self-made satisfaction table was used to evaluate the results of the students, which mainly assessed the effect of teaching methods, self-growth, enthusiasm and other aspects. The students’ satisfaction was investigated in the form of a questionnaire, and the questionnaire results were statistically divided into 3 levels. Scores ≤ 60 was not satisfied, 61 ~ 80 was generally satisfied, 81 ~ 100 was very satisfied. Satisfaction rate = (generally satisfied + very satisfied)/total number of students ×100.00%.

### Statistical analysis

SPSS 20.0 software was implemented to analyze the data in this study. Counting data were represented by [*n* (%)] and *χ*^2^ test was performed for comparison. Measurement data were represented by (*x* ± *s*) and t test was performed for comparison. Effect sizes (Cohen’s *d*) and 95% confidence intervals (CI) were calculated. *p* < 0.05 meant the difference was statistical significance.

## Results

### General data in 2 groups

The PBL group included 27 males and 30 females. The average age was (30.12 ± 0.54) years, ranging from 25 to 35 years. The LBL group included 25 men and 32 women. The average age was (30.06 ± 0.48) years, ranging from 24 to 35 years. No significant difference was discovered in age and gender between the two groups (*p* > 0.05), suggesting the general data between the two groups were comparable.

### Teaching effect in 2 groups

**Figure 1:**
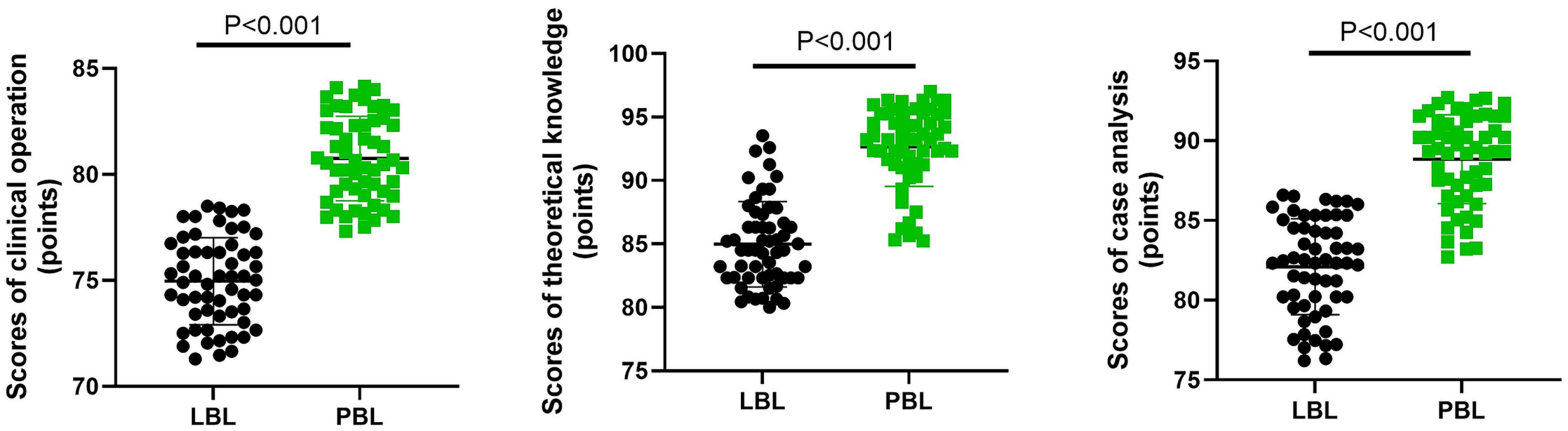
Comparison of teaching effect between the two groups. Each point in the figure represented the score of the corresponding group, and the error bars indicated the mean ± standard deviation, which was used to reflect the dispersion of the data. ^**^p < 0.01.

Compared with the LBL group, the PBL group has significant differences in clinical operation, theoretical knowledge, and case analysis scores (*p* < 0.001, Figure 1). Specifically, in the clinical operation score, the mean difference between the PBL group and the LBL group was 5.78 points, with a 95% CI of (5.031–6.533) and a Cohen’s d of −2.853; in the theoretical knowledge score, the mean difference was 7.67 points, with a 95% CI of (6.464–8.877) and a Cohen’s d of −2.365; in the case analysis score, the mean difference was 6.74 points, with a 95% CI of (5.664–7.817) and a Cohen’s d of −2.326. These results suggested that compared with the LBL teaching method, the PBL teaching method was conducive to improving the teaching effectiveness of students.

### Student satisfaction in 2 groups

The student satisfaction of the LBL group was 85.96% (49/57), and that of the PBL group was 96.49% (55/57). In contrast to the LBL group, the student satisfaction in the PBL group was higher (*p* = 0.047, 95% CI = 0.437–0.991, Table 1). These results suggested that compared with the LBL teaching method, the PBL teaching method was conducive to improving the satisfaction of students.

**Table 1:** Student satisfaction in two groups.

Groups	*n*	Very satisfied	Generally satisfied	Dissatisfied	Total satisfaction rate
LBL	57	25	24	8	49 (85.96%)
PBL	57	30	25	2	55 (96.49%)
*χ* ^2^					3.946
*P*					0.047
95% CI					0.437–0.991

## Discussion

The traditional LBL teaching is mainly a subject-based, teacher-centered, and classroom-taught teaching method (11). In this teaching method, teachers directly impart relevant knowledge to students during the teaching process, while the students remain in a passive state of receiving information (12). As a long-standing dominant teaching model, LBL teaching can disseminate knowledge within a limited time and help students form deep memories (6). However, the drawbacks of this teaching model are also obvious. Firstly, there is little interaction and communication between the teacher and the students in the classroom, and the students are in a passive state of listening and mechanical memorization. The teacher neglects to cultivate students’ ability to apply knowledge and innovative thinking (9). Secondly, students regard rote memorization and passing exams as the purpose of learning, which makes it difficult for them to exert their subjective initiative in learning, and thus they are likely to be cultivated into “high scores but low abilities” students (13). Therefore, how to change the traditional teaching mode and effectively cultivate students’ ability to analyze and solve problems is particularly important. Compared with other teaching methods, PBL pays more attention to the cultivation of students’ practical ability. Students can actively participate in the teaching process, which promotes communication between teachers and students and effectively stimulates students’ learning enthusiasm (14).

In our study, the results suggested that in contrast to the LBL group, the scores of clinical operation, theoretical knowledge and case analysis and the student satisfaction in the PBL were higher. This indicated that PBL teaching model had a significant effect in the teaching of respiratory critical care medicine, helping to improve the examination scores of interns and gaining the recognition of students. This is because that PBL teaching method introduces cases to students to pose relevant questions, and students construct knowledge around these questions, thereby effectively stimulating students’ learning enthusiasm and cultivating their ability to analyze and solve problems (15). Furthermore, the PBL teaching method focuses on enhancing the clinical thinking ability, practical operation ability, diagnosis and treatment ability of interns, thereby effectively improving the learning efficiency and cultivating the learning ability, innovation ability and practical ability of interns. Therefore, it is more likely to be accepted by interns (16).

There are some limitations of our study. First, our sample size is relatively small. From a statistical perspective, a small sample size can lead to an increase in sampling error, making the selected sample unable to accurately represent the overall characteristics of the population. For instance, when evaluating the effect of PBL teaching methods on the improvement of clinical operational skills, due to the limited sample size, it may not cover different student groups with varying learning abilities, knowledge bases, and operational styles. Some students with special learning characteristics or operational advantages may not have been fully included, resulting in a deviation in the estimation of the effectiveness of PBL teaching methods, possibly overestimating or underestimating their actual impact. Furthermore, in data analysis, small samples may fail to accurately capture some subtle differences. When comparing the differences in theoretical knowledge acquisition between the PBL group and the LBL group, there might be some potential factors that have significant impacts on the teaching effect. However, due to the insufficient sample size, these factors were not fully reflected in the data, thereby affecting the precise judgment of the differences in the effectiveness of theoretical knowledge transmission between the two teaching methods. Second, in this study, due to the particularity of the teaching model, it was difficult to conduct a blind test on both the students and the instructors. This may lead to the following several deviations: (1) Students have expectations regarding the teaching methods of their respective groups, and these expectations can significantly alter their learning attitudes and performance. For students in the PBL group, they may expect that the PBL teaching method is more innovative and interactive, thus engaging in learning with a more positive attitude, actively participating in problem discussions and case analyses, and demonstrating greater initiative and exploration spirit in clinical operations and case analyses. On the contrary, students in the LBL group may consider the lecturing method to be more traditional and passive, resulting in relatively lower learning enthusiasm. During the learning process, they rely more on the teacher’s explanations and lack active thinking and in-depth exploration. This difference in attitude caused by expectations may further affect the learning outcome, making it difficult for the research results to accurately reflect the true differences between the two teaching methods; (2) during the teaching process, teachers may become aware of the teaching model they have adopted and unconsciously adjust their teaching behaviors and attitudes. Teachers who adopt the PBL teaching method may, because they recognize that this method emphasizes students’ autonomous learning and problem-solving abilities, pay more attention to guiding students to think, encouraging them to ask questions, and giving them more autonomy in the teaching process. While teachers who adopt the lecture-based teaching method may, due to their habit of traditional teaching methods, pay more attention to the systematic and comprehensive explanation of knowledge after realizing the teaching method, but may be lacking in interaction and student participation. Such changes in teachers’ teaching behaviors and attitudes may not be entirely determined by the teaching method itself, but are influenced by their understanding of the teaching method, which may interfere with the accurate assessment of the effectiveness of the two teaching methods; (3) Although this study adopted a blind evaluation method, in the actual teaching process, students and teachers were aware of the groupings. When communicating with the evaluators or presenting their learning outcomes, they might exhibit different attitudes and behaviors. Students in the PBL group might be more confident in this new teaching method and be more proactive when communicating with the evaluators, elaborating on their thoughts and gains during the problem-solving process, and demonstrating stronger communication skills and self-confidence. While students in the LBL group might show relatively more conservative behavior when presenting their learning outcomes. Similarly, teachers, when communicating with the evaluators, might present different attitudes and expressions due to differences in their understanding and implementation of the teaching methods. These different attitudes and behaviors might indirectly affect the evaluators’ judgments, causing deviations in the assessment results and preventing them from objectively and accurately reflecting the actual effectiveness of the two teaching methods. Third, this study was conducted in a single center, which severely limits the general applicability of the research results. There are significant differences in medical resources, teaching facilities, faculty quality, and patient populations among different hospitals, and these factors can all have a significant impact on teaching outcomes. For instance, in terms of medical resources, some large comprehensive hospitals may possess more advanced medical equipment and technologies, providing students with more opportunities to encounter complex cases and access cutting-edge treatment methods. This might help enhance students’ abilities in clinical operations and case analysis. However, smaller hospitals or specialized hospitals may have relatively limited resources, and the types of cases and operational opportunities that students encounter during their studies might be more limited. In terms of teaching facilities, there are differences among different hospitals in terms of classroom environments, simulation teaching equipment, etc. Hospitals that are equipped with advanced simulation operating rooms and teaching software can provide students with a more realistic clinical operation simulation environment, which is conducive to students’ better mastery of clinical skills. However, hospitals with poor teaching facilities may not be able to offer the same high-quality learning conditions, which will affect students’ learning outcomes. The quality of teaching staff is also a key factor influencing the teaching outcome. Teachers in different hospitals vary in terms of clinical experience, teaching ability, and the application of teaching methods. Experienced and highly competent teachers can better guide students in their learning, stimulate their interest and potential in study, while teachers with relatively weaker teaching abilities may not achieve the same teaching effect. Furthermore, the differences among patient groups will also have an impact on teaching. The types of diseases, severity and complexity of conditions of patients admitted to different hospitals vary, and the characteristics of cases that students encounter during their studies also differ. This may lead to differences in students’ understanding and handling abilities of diseases after they study in different hospitals. Therefore, the results obtained from a single-center study may not be applicable in other hospitals or teaching environments. Further multi-center research is needed to verify and promote these findings.

In conclusion, the PBL teaching method is conducive to improving the teaching effectiveness and increasing the satisfaction of students. It has high promotion and application value.

## Data Availability

The datasets presented in this study can be found in online repositories. The names of the repository/repositories and accession number(s) can be found in the article/supplementary material.
